# Impact of Internet Integrated Financial Education on Students’ Financial Awareness and Financial Behavior

**DOI:** 10.3389/fpsyg.2021.751709

**Published:** 2021-09-28

**Authors:** Hong-Cheng Liu, Jie-Shin Lin

**Affiliations:** Department of Public Policy and Management, I-Shou University, Kaohsiung, Taiwan

**Keywords:** internet, financial education, financial awareness, financial behavior, knowledge experience

## Abstract

In daily life, most people engage in money-related behavior. Adequate financial knowledge is required to successfully manage tasks, such as daily expenditure and the transformation of assets or debts, small, or large. However, the extent of financial knowledge may vary between individuals. With inadequate financial knowledge, people may easily fall into financial difficulties without having sufficient knowledge to redress them. A total of 217 students from departments of finance in universities in Fujian completed an 18-week educational course delivered *via* the Internet on integrated financial education (5h per week for a total of 90h). The conclusions were as follows: (1) The Internet can be used to provide education on making ends meet, cutting costs, and increasing profits. It is suitable for beginner students and new graduates who are rapidly accumulating money management experience. (2) Knowledge provided in the course includes the causes of investment, comprehensive changes in the market, unexpected risks, and wrong decision-making. As such, education provided through the Internet can assist in the teaching of money management and investment. (3) Providing teaching on integrated financial education through the Internet avoids the pitfalls of getting lost in the real-world investment market. We expected to cultivate students’ finance-related knowledge, skills, and attitudes through internalization of the financial literacy of money management.

## Introduction

Equal importance should be given to financial education as already given to language, mathematics, sciences, and humanities. Money-related behavior is for most an essential component of life, ranging from the management of daily expenses to selling and buying large financial assets. Management of both assets and debts requires financial knowledge, but for some this knowledge may be inadequate. When financial knowledge is inadequate, people can easily fall into financial difficulties without the knowledge or insight required to address these difficulties.

A key factor of poverty is having imbalanced finances or “not making ends meet.” To solve this problem, financial education can be used to guide individuals in acquiring wealth through financial management, with the central idea of “creation.” Financial pressure caused by excessive consumption could be reduced by adjusting the concepts of income and expenditure through the consideration of items and labor. Surplus could be directed toward suitable investment using risk control to increase income and to gradually increase non-labor income through such investment tools. In the early stages, this could be the income source for the family who can gradually achieve financial freedom and alleviate poverty by using their non-labor income to pay all their expenses. People stress about financial freedom because the value would be enhanced when they do not directly work for money.

In the current study, the Internet was applied to study the correlations between financial education, student financial cognition, and financial behavior. We expected to cultivate and change students’ existing concepts of income and expenditure, establish a better understanding of assets, debts, and cash flow, adjust students’ personal financial structures, and reinforce awareness of risk management in investment.

## Literature Review

Scherer et al. (2019) discussed different teaching methods for delivering financial education and the effectiveness of these. He showed that financial education can positively enhance students’ financial knowledge. An interview with teachers after completing a course also revealed an important role for the cognition of financial education and enhancement of the understanding of the financial education curriculum. Gentina et al. (2018) demonstrated that people with financial education can show a final wealth difference up to 30–40%. In other words, an individual can acquire short-term and long-term real gains through financial education.

Another study demonstrated that people without financial education or training are more likely to lose in the investment market. It is common for people lacking the basis of financial education and literacy to accumulate many experiences of failed investment, including high borrowing costs or increasing unnecessary expenses, rather than cultivating long-term and stable investment habits.

Hanson and Olson (2018) indicated that financial cognition broadly affects financial behavior, and as such the enhancement of financial cognition provides significant improvements in personal financial management. Taking the example of pension planning, financial cognition has been found to be an important factor in people engaging in financial behavior and experiencing benefits from financial services. In particular, Hert et al. (2012) stated people living in urban areas can acquire various saving products, financing products, and financial loan channels. Nevertheless, the middle classes tend not to effectively use such financial services, primarily due to a lack of financial cognition. Moreno-herrero et al. (2018) revealed that modern people’s financial behavior does not lead to the most appropriate financial decisions, including those relating to mortgage loans, the planning of loan repayments, the financial terms required for retirement, property mortgages, chattel mortgages, and interest payments, and the repayment of principal.

Looking at the financial socialization of college freshmen, Moreno-herrero et al. (2018) analyzed the role of parents, past working experiences, and learning experiences in financial education at school with structural equation modeling. The researchers found that three factors could predict financial behavior. Parents’ past working experiences together with the quality of their relationship and communication with their children, and having learning experience in financial education at school, presented the greatest benefits.

Brent and Ward (2018) proposed that inadequate financial behavior would induce people to enhance their financial knowledge. Therefore, the effectiveness of financial education depends on previous financial behavior and insight into that financial behavior. Granić and Marangunić (2019) indicate that current research stresses the effect of financial education on financial decisions and the poor population in developing countries based on random control. Their results reveal that effective financial education leads to improvement in financial behavior.

## Hypothesis

From the above literature, the following hypotheses were inferred.

H1: There are positive relationships between financial education and financial cognition.

H1-0: Financial education does not show a significant and positive relationship with financial cognition.
H1-1: Financial education reveals a remarkable and positive relationship with financial cognition.
H2: There will be positive relationships between financial cognition and financial behavior.
H2-0: Financial cognition does not appear a notable and positive relationship with financial behavior.
H2-1: Financial cognition reveals a significant and positive relationship with financial behavior.
H3: There will be a positive relationship between financial education and financial behavior.
H3-0: Financial education does not present a notable and positive relationship with financial behavior.
H3-1: Financial education shows a remarkable and positive relationship with financial behavior.

## Materials and Methods

### Operational Definitions

#### Financial Education

Chou and Chan (2018) proposed that financial education consists of three dimensions: degree of awareness, knowledge experience, and ability practice.

#### Financial Cognition

Lusardi (2019) proposed that financial cognition contains the following dimensions: cutting costs and increasing profits, investment decisions, and good habits.

#### Financial Behavior

Miller and Xu (2019) proposed that financial behavior should be measured with a single dimension.

### Research Sample and Questionnaire

A total of 360 questionnaires were distributed to students in departments of finance in universities in Fujian. A total of 265 valid copies were returned, representing a retrieval rate of 74%.

### Reliability and Validity Testing

Validity refers to the extent that a measurement tool measures what the researcher really wants to measure. The assessment of validity generally comprises the evaluation of content validity, criterion-related validity, and construct validity. The questionnaire items used in this study were taken from those used in previous domestic and international studies, suggesting that the questionnaire holds certain content validity.

The overall structural causal relationship results revealed that the overall model fit reached a reasonable range in demonstrating favorable convergent validity and predictive validity. Item-to-total correlation coefficients were used to test the construct validity of the questionnaire. The correlation coefficients were all higher than 0.7, revealing a certain degree of construct validity.

To further understand the reliability and validity of the questionnaire, Cronbach’s αs were calculated. As higher αs reflect better reliability, the measured Cronbach’s α in this study of 0.75–0.90 reflects acceptable to excellent reliability.

## Results

### LISREL Indicator

The research data are presented in Table 1. The preliminary fit, internal fit, and overall fit of the model are explained as follows.

**Table 1:** Linear structural model analysis.

Evaluation item	Parameter/Evaluation standard		Result	*t*
Preliminary fit	Financial education	Degree of awareness	0.714	9.62^**^
		Knowledge experience	0.736	11.16^**^
		Ability practice	0.757	12.62^**^
	Financial cognition	Cutting costs and increasing profits	0.722	10.43^**^
		Investment Decision-making	0.743	11.87^**^
		Good habits	0.762	14.58^**^
Internal fit	Financial education→financial cognition		0.846	31.56^**^
	Financial cognition → financial behavior		0.875	47.15^**^
	Financial education → financial behavior		0.825	23.77^**^
Overall fit	*X^2^*/*df*		1.637	
	GFI		0.964	
	AGFI		0.931	
	RMR		0.005	

** *p*<0.01.

Table 1 shows that the three dimensions of financial education (degree of awareness, knowledge experience, and ability practice) could significantly explain financial education (*t*>1.96, *p*<0.05). The three dimensions of financial cognition (cutting costs and increasing profits, investment decision-making, and good habits) could remarkably explain financial cognition (*t*>1.96, *p*<0.05), and the single factor of financial behavior notably explained financial behavior (*t*>1.96, *p*<0.05). Thus, the overall model presented a good preliminary fit.

In terms of internal fit, financial education was positively and significantly correlated with financial cognition (0.846, *p*<0.01). Financial cognition was positively and significantly correlated with financial behavior (0.875, *p*<0.01), and financial education was significantly and positively correlated with financial behavior (0.825, *p*<0.01). Therefore, H1, H2, and H3 were supported.

The overall model fit standards, *χ*^2^/*df*=1.637, were smaller than the standard 3, and RMR=0.005 revealed the proper results of *χ*^2^/*df* and RMR. Furthermore, Chi-square is sensitive to sample size, and therefore, it is not suitable for directly judging the model fit. However, the overall model fit standards (GFI=0.964 and AGFI=0.931) reached the standard 0.9 (the closer GFI and AGFI are to 1, the better the model fit) such that the model presented favorable fit indices.

The research results in Table 2 reveal a notable and positive relationship between financial education and financial cognition (0.846**) that H1-0 is rejected and H1-1 is accepted. H1 is therefore supported. Financial cognition appears a significant and positive relationship with financial behavior (0.875**) that H2-0 is rejected and H2-1 is accepted. Apparently, H2 is supported. Financial education reveals a positive relationship with financial behavior (0.825**) that H3-0 is rejected and H3-1 is accepted. Accordingly, H3 is supported.

**Table 2:** Hypothesis tests.

Research hypothesis	Correlation	Empirical result	*p*	Result
H1	+	0.851	*p* <0.01	Supported
H2	+	0.824	*p* <0.01	Supported
H3	+	0.836	*p* <0.01	Supported

## Discussion

Financial education can be provided by teachers or professional teachers through a combination of systematic teaching and the application of materials, and teaching aids to advance education, knowledge, and skills in using money or resources. Nowadays, money is not referred to as the representation of money in general but is one of the value exchange tools. Stocks, funds, futures, insurance, automobiles, jewels, real estate, and enterprises are valuable; they are the extension of commodities. Financial education, therefore, is the education of exchange ability. By schools planning school-wide or teachers designing class financial education activities, the design of integrating integrated curricula into teaching or applying flexible time to the practice could enhance the extension of financial education curricula and avoid time shortage. Besides, Fox et al. (2005) mentioned that well applying teaching resource websites for financial education could help teachers to effectively promote financial education and financial literacy. Insisting on the idea of resource sharing to establish the material sharing platform in schools, the teaching content could be expanded and updated at any time to enrich teachers’ financial literacy. Moreover, the teaching content should be combined with students’ real-life experience to cultivate students’ financial behavior and stress on the practicality in life to induce students’ learning interests and teach students financial knowledge and financial behavior. Fernandes et al. (2014) in his correlational studies that measure financial literacy finds stronger associations with financial behaviors. Teachers, on the other hand, should well-utilize various channels as well as actively and positively enhance personal financial education knowledge and skills. In addition to study activities conducted by education-related units, teachers could participate in activities help by private groups and banks, read books, newspaper, and magazines, browse the Internet, and converse with professional peers to absorb financial knowledge and enhance personal financial literacy.

## Conclusion

The current findings show that financial education can enhance people’s financial knowledge, improve people’s ability to comprehend investment markets, teach people how to diagnose personal financial health, and cultivate a lifelong interest in the study of money. Financial statement is the most important issue in financial management. People generally agree with the importance of financial reports, but most people would give up due to the dullness and complication. Financial education creates personal financial statement covering balance sheet, income statement, and simple cash flow statement for people learning the process to make financial reports for further application to daily life so as to reduce pressure of making financial reports. In other words, people who have obtained a basic financial education may more readily comprehend financial cognition and not get lost in their financial behavior. Applying money and/or capital to financial investment with inadequate financial knowledge or financial cognition would lead to difficulty in controlling the risks in the investment market and increase the likelihood of money loss. Although financial education is emphasized in various industries, the concept of financial education is currently still only theoretical. The provision of a simulated financial management model with the characteristics of educational entertainment for students learning about financial statements and obtaining related financial knowledge and skills until now has been scarce. Financial education cultivates students’ educational spirit of building the ability of digital management of financial structure, carefully planning the adjustment of income and expenditure in financial statements, and authentically experiencing the situations of living within the means and cutting costs and increasing profits. It is the education suitable for beginners or fresh graduates rapidly accumulating financial experiences.

Learning with the assistance of tutors and teachers would present better learning effectiveness. The systematic cultivation of teachers could rapidly establish improved concepts of money, enhance national financial cognition and ability, change financial behavior and enhance investment effectiveness, reduce the wealth gap caused by inadequate knowledge, and further move toward the goals of national financial health and average prosperity.

## Data Availability Statement

The original contributions presented in the study are included in the article/supplementary material, further inquiries can be directed to the corresponding author.

## Ethics Statement

The research protocol was approved by the Ethical Committee of the I-Shou University, Taiwan. Written informed consent was obtained from all the participants.

## Author Contributions

H-CL performed the initial analyses and wrote the manuscript. J-SL assisted in the data collection and data analysis. All authors contributed to the article and approved the submitted version.

## Conflict of Interest

The authors declare that the research was conducted in the absence of any commercial or financial relationships that could be construed as a potential conflict of interest.

## Publisher’s Note

All claims expressed in this article are solely those of the authors and do not necessarily represent those of their affiliated organizations, or those of the publisher, the editors and the reviewers. Any product that may be evaluated in this article, or claim that may be made by its manufacturer, is not guaranteed or endorsed by the publisher.
